# Herb-drug interaction between Echinacea purpurea and darunavir/ritonavir in HIV-infected patients

**DOI:** 10.1186/1758-2652-13-S4-P187

**Published:** 2010-11-08

**Authors:** J Moltó, M Valle, C Miranda, S Cedeño, E Negredo, MJ Barbanoj, B Clotet

**Affiliations:** 1 Hospital Universitari Germans Trias i Pujol, “Lluita contra la Sida” Foundation, HIV Clinic, Badalona, Spain; 2 Institut de Recerca de l'Hospital de la Santa Creu i Sant Pau, PK/PD modelling and simulation, Barcelona, Spain; 3 Hospital Universitari Germans Trias i Pujol, "IrsiCaixa" Foundation, HIV Clinic, Badalona, Spain; 4 Institut de Recerca de l'Hospital de la Santa Creu i Sant Pau, Centre d'Investigació del Medicament, Barcelona, Spain

## Purpose of the study

To investigate the potential of a commonly used botanical supplement, Echinacea purpurea, to interact with the boosted protease inhibitor darunavir/ritonavir.

## Methods

Open-label, fixed-sequence study in 15 HIV-infected patients receiving antiretroviral therapy including darunavir/ritonavir (600/100 mg twice daily) for at least 4 weeks. Echinacea purpurea root extract-containing capsules were added to the antiretroviral treatment (500 mg every 6 hours) from days 1 to 14. Darunavir concentrations in plasma were determined by using HPLC immediately before and 1, 2, 4, 6, 8, 10 and 12 hours after a morning dose of darunavir/ritonavir on days 0 (darunavir/ritonavir) and 14 (darunavir/ritonavir + echinacea). Individual darunavir pharmacokinetic parameters were calculated by using non-compartmental analysis, and were compared between days 0 and 14 by using the geometric mean ratio (GMR) and its 95% confidence interval (95% CI).

## Results

Median (range) age was 49 (43-67) years, and body mass index was 24.2 (18.7-27.5) kg/m^2^. Echinacea was well tolerated and all participants completed the study. Relative to administration of darunavir/ritonavir alone, its coadministration with Echinacea purpurea resulted in little change in darunavir pharmacokinetic parameters. Table 1

**Table 1:** 

	DRV/r	DRV/r + Echinacea	GMR (95% CI)	p
Cτ (ng/mL)	2.1 (1.6-2.7)	1.7 (1.4-2.2)	0.84 (0.59-1.19)	0.311

AUCτ (ng.h/mL)	46.2 (39.0-54.7)	41.6 (35.1-49.2)	0.90 (0.71-1.14)	0.374

Cmax (ng/mL)	6.4 (5.5-7.4)	6.2 (5.3-7.25)	0.98 (0.79-1.21)	0.810

## Conclusions

coadministration of Echinacea purpurea with darunavir/ritonavir was safe and well tolerated in HIV-infected patients; data suggest that no dose adjustment for darunavir/ritonavir is necessary.

